# Diffusion MRI Characteristics After Concurrent Radiochemotherapy Predicts Progression-Free and Overall Survival in Newly Diagnosed Glioblastoma

**DOI:** 10.18383/j.tom.2015.00115

**Published:** 2015-09

**Authors:** Warren Chang, Whitney B. Pope, Robert J. Harris, Anthony J. Hardy, Kevin Leu, Reema R. Mody, Phioanh L. Nghiemphu, Albert Lai, Timothy F. Cloughesy, Benjamin M. Ellingson

**Affiliations:** Departments of 1 Radiological Sciences,; 2 Biomedical Physics,; 3 Neurology, and; 4 Psychiatry and Biobehavioral Sciences, David Geffen School of Medicine;; 5 Department of Bioengineering, Henry Samueli School of Engineering and Applied Science; and; 6 Neuro-Oncology Program, University of California, Los Angeles, Los Angeles, CA

**Keywords:** glioblastoma, ADC histogram analysis, diffusion MRI

## Abstract

The standard of care for newly diagnosed glioblastoma (GBM) is surgery first, radiotherapy (RT) with concurrent temozolomide (TMZ) second, and adjuvant TMZ last. We hypothesized patients with low diffusivity measured using apparent diffusion coefficient (ADC) histogram analysis evaluated after RT + TMZ and before adjuvant TMZ would have a significantly shorter progression-free survival (PFS) and overall survival (OS). To test this hypothesis, we evaluated 120 patients with newly diagnosed GBM receiving RT + TMZ followed by adjuvant TMZ. Magnetic resonance imaging was performed after completing RT + TMZ and before initiating adjuvant TMZ. A double Gaussian mixed model was used to describe the ADC histograms within the enhancing tumor, where ADC_L_ and ADC_H_ were defined as the mean ADC value of the lower and higher Gaussian distribution, respectively. An ADC_L_ value of 1.0 μm^2^/ms and ADC_H_ value of 1.6 μm^2^/ms were used to stratify patients into high- and low-risk categories. Results suggested that patients with a low ADC_L_ had a significantly shorter PFS (Cox hazard ratio = 0.12, *P* = .0006). OS was significantly shorter with low ADC_L_ tumors, showing a median OS of 407 versus 644 days (Cox hazard ratio = 0.31, *P* = .047). ADC_H_ did not predict PFS or OS when accounting for age and ADC_L_. In summary, after completing RT + TMZ, newly diagnosed glioblastoma patients with a low ADC_L_ are likely to progress and die earlier than patients with a higher ADC_L_. ADC histogram analysis may be useful for patient-risk stratification after completing RT + TMZ.

## Introduction

Glioblastoma (GBM) is the most common and deadly form of primary brain tumors in adults. The current standard—aggressive therapy consisting of maximal surgical resection followed by concurrent radiotherapy (RT), temozolomide (TMZ) chemotherapy, and adjuvant TMZ—has shown a median survival of only 14.6 months (1–3). Although GBMs generally have a very poor prognosis, there are clearly cohorts of patients that benefit from specific therapies. Thus, there is great interest in identifying risk factors and biomarkers for predicting response to therapy beforehand. Patient age at diagnosis, neurological performance status, extent of surgical resection, radiographic composition of the tumor, tumor volume and location, isocitrate dehydrogenase 1 mutation status, gene expression subtype, and O6-methylguanine methyltransferase promoter methylation are commonly assessed prognostic characteristics for GBM (4–12).

The use of imaging features to phenotype tumors and to predict therapeutic response is an attractive option compared with more invasive approaches based on tissue-derived biomarkers. By noninvasively characterizing the composition of the tumor microenvironment, features associated with particular response patterns can be identified that lead to the potential for patient cohort enrichment for use in clinical trials. We recently showed that the apparent diffusion coefficient (ADC) characteristics measured using diffusion magnetic resonance imaging (MRI) techniques can be used to predict both progression-free survival (PFS) and overall survival (OS) in GBM patients treated with bevacizumab at recurrence (13–15). Specifically, results in both single and multicenter trials have shown high ADC measurements within the contrast-enhancing tumor regions predict a favorable response to bevacizumab treatment at recurrence as indicated by a longer PFS and OS, whereas patients with low ADC measurements have a significantly shorter PFS and OS. It is important to note that results have also suggested that the prognostic capabilities of ADC measurements may be specific to bevacizumab therapy at recurrence, because no difference in PFS or OS were noted in bevacizumab-naïve patients treated with chemotherapy at recurrence (13). However, it is conceivable that ADC measurements may also be prognostic when used to evaluate the phase of adjuvant TMZ before the first recurrence, because various studies have suggested a general increase in ADC after successful RT + TMZ (16–21).

In this study, we examined a cohort of 120 patients with a newly diagnosed GBM that underwent tumor resection followed by RT + TMZ. We then evaluated the diffusion MRI characteristics within the tumor 4 weeks after completing RT+TMZ—just before starting the adjuvant phase of TMZ therapy. We hypothesized that high ADC measurements within contrast-enhancing voxels after completing RT + TMZ would indicate a longer PFS and OS.

## Methodology

### Patient Characteristics

All patients participating in this study signed institutional review board-approved informed consent. Data acquisition was performed in compliance with all applicable Health Insurance Portability and Accountability Act regulations. Patients were retrospectively selected from our institution's neuro-oncology database. Initially, a total of 169 patients who met the following criteria were selected: (1) pathology-confirmed glioblastoma, (2) treatment with standard external beam radiotherapy and concurrent TMZ followed by adjuvant TMZ, and (3) MRI scans obtained after surgical resection and within 4 weeks after RT + TMZ—just before the adjuvant phase of TMZ. The average age for this population was 58.4 years (±11-year SD), the average Karnofsky performance status score was 86 (±10 SEM), and 57% of the patients were male (97/169). In total, 70 patients had a gross total resection at the time of initial surgery, 73 had a subtotal resection, and 26 had only a biopsy before radiochemotherapy.

Of all patients enrolled, 120 had good-quality diffusion-weighted images and were included in the final analyses for this study. Exclusions were based on gross geometric distortions or low signal-to-noise ratios in the raw diffusion-weighted imaging datasets or patients with a contrast-enhancing tumor less than 0.1 cc as seen on the first MRI scan after RT + TMZ. These follow-up scans were obtained approximately 10 weeks from the time of treatment initiation (mean = 75 ± 2.6-day SEM) or approximately 4 weeks from the end of initial radiochemotherapy. At the time of last assessment, 104 of the 120 patients had died.

### Treatment Paradigm

**Figure 1.**
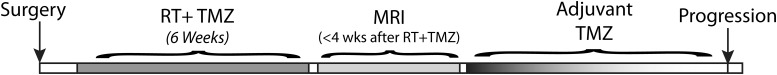
Treatment and MRI evaluation timeline. Patients were treated with 60 Gy of external beam radiation therapy (2-Gy fractions given once daily for 5 days over a 6-week period) with concomitant TMZ (75 mg/m^2^ orally or intravenously for 42 consecutive days), followed by a 28-day break and then the start of adjuvant TMZ (150 mg/m^2^ orally or intravenously for 5 consecutive days during the first 28-day cycle, followed by 200 mg/m^2^ orally or intravenously for 5 consecutive days during the first 28-day cycle for a maximum of 6 cycles). Diffusion and standard anatomical MRI were performed within 10 weeks after the start of RT + TMZ or within 4 weeks from the end of RT + TMZ—just before adjuvant TMZ.

Patients were treated with 60 Gy of external beam radiation therapy (2-Gy fractions given once daily for 5 days over a 6-week period) with concomitant TMZ (75 mg/m^2^ orally or intravenously for 42 consecutive days), followed by a 28-day break, and then adjuvant TMZ (150 mg/m^2^ orally or intravenously for 5 consecutive days in the first 28-day cycle followed by 200 mg/m^2^ orally or intravenously for 5 consecutive days in the first 28-day cycle for a maximum of 6 cycles). Diffusion and standard anatomical MRI were performed within 10 weeks after the start of RT + TMZ or within 4 weeks from the end of RT + TMZ—just before adjuvant TMZ (Figure 1). The beginning of adjuvant TMZ and the MRI evaluation were performed on the same day. This is typically the first imaging evaluation after completing RT + TMZ and is therefore an important clinical decision-making time point.

### MRI

Diffusion and structural MRIs were obtained on a GE Signa Excite HDx or Lx 1.5T (GE Healthcare, Waukesha, WI); Siemens Avanto or Sonata 1.5T (Siemens Healthcare, Erlangen, Germany); or Siemens Trio, Allegra, or Verio 3T MRI system. Standard anatomical MRI consisted of pre- and postcontrast (gadolinium-diethylenetriamine pentacetic acid at a dose of 0.1 mmoL/kg body weight; Magnevist, Bayer Schering Pharma, Leverkusen, Germany) axial T1-weighted images along with precontrast axial T2-weighted and fluid-attenuated inversion recovery sequences with standard sequence parameters. Patients also received diffusion-weighted images with an echo/repetition time = 80 to 120 ms/>5000 ms, matrix size = 128 × 128, slice thickness = 3 mm with no interslice gap, and *b* values of 0 and 1000 s/mm^2^ in 3 orthogonal directions. ADC maps were calculated for each image voxel as ADC(*x*, *y*, *z*) = −1/1000 · ln[*S*(*x*, *y*, *z*)/*S*_0_(*x*, *y*, *z*)], where *S*(*x*, *y*, *z*) is the signal intensity of the voxel at coordinate (*x*, *y*, *z*) with *b* = 1000 s/mm^2^ and *S*_0_(*x*, *y*, *z*) is the signal intensity at voxel (*x*, *y*, *z*) with *b* = 0 s/mm^2^.

### ADC Histogram Analysis

**Figure 2.**
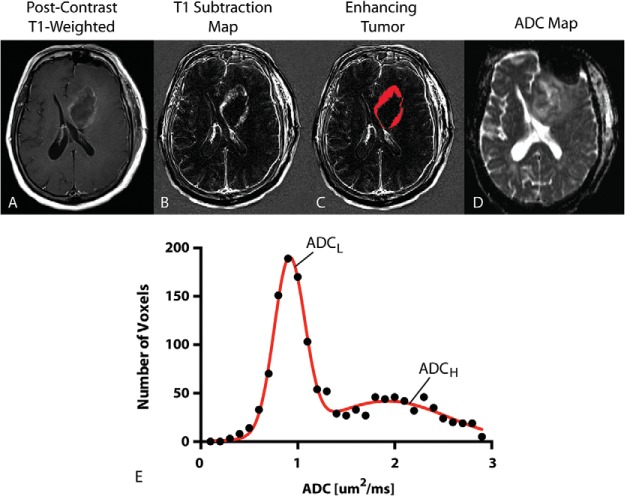
ADC histogram analysis. (A) Postcontrast T1-weighted image showing ring-enhancing tumor in the left frontal lobe. (B) T1 subtraction map generated from subtracting precontrast from postcontrast T1-weighted images. (C) Enhancing tumor mask extracted from T1 subtraction maps. (D) ADC map used for diffusion MRI phenotyping. (E) Resulting ADC histogram (raw data, black closed circles; double Gaussian mixed model fit, solid red line). ADC_L_ = mean of the lower Gaussian distribution estimated the double Gaussian mixed model fit. ADC_H_ = mean of the higher Gaussian distribution estimated from the double Gaussian mixed model fit.

ADC histogram analysis was performed using previously described techniques (13–15). Briefly, contrast-enhancing tumor regions were segmented on T1 subtraction images calculated by subtracting precontrast from postcontrast T1-weighted images (22). ADC characteristics from within enhancing regions were then extracted. A double Gaussian mixed model was used to describe the ADC histogram using nonlinear regression, where ADC_H_ reflects the mean ADC in the larger of the two Gaussian distributions and ADC_L_ is the mean ADC value of the lower Gaussian distribution (Figure 2). Both ADC_L_ and ADC_H_ were used as the primary imaging biomarker for the current study using GraphPad Prism version 6 (GraphPad Software, Inc., La Jolla, CA).

### Definition of Tumor Progression

Progression was defined prospectively by the treating neuro-oncologists if subsequent scans showed an increase in imaging-evaluable tumor (≥25% increase in the sum of enhancing lesions, new enhancing lesions > 1 cm^2^, an unequivocal qualitative increase in nonenhancing tumor, or an unequivocal new area of noncontrast-enhancing tumor). Patients were required to have a stable or decreasing contrast agent dose before partial or complete response could be determined. In addition, patients who required an increased dosage of steroids to maintain neurologic function, even when anatomical images showed no worsening, were considered to be stable but required early reevaluation. Patients who experienced significant neurologic decline were also declared to have progressed at the time of irreversible decline. Landmark PFS was defined as the time between the MRI scan following completion of RT + TMZ and progression. Landmark OS was defined as the time between the MRI scan and death.

### Statistical Analyses

Receiver operating characteristic (ROC) analysis was used to determine whether a low ADC_L_ could identify patients who progressed within 6 months from starting adjuvant TMZ (i.e., PFS6) and patients who died within 12 months from starting adjuvant TMZ (e.g., OS12) using area under the ROC curve (AUC) as a measure of performance. An ADC_L_ value of 1.0 μm^2^/ms and ADC_H_ value of 1.6 μm^2^/ms were chosen as the primary biomarkers of interest because these values were near the median of the patient distribution and found to have the highest likelihood ratio (sensitivity/[1 − specificity]) for both PFS6 and OS12. This cutoff was then used to stratify PFS and OS using both log-rank analysis on Kaplan-Meier data and multivariate Cox regression analysis using age as an additional covariate. A *P* value less than .05 was considered statistically significant, and a *P* value less than .10 was considered trending toward significance.

## Results

### ROC Analysis

**Figure 3.**
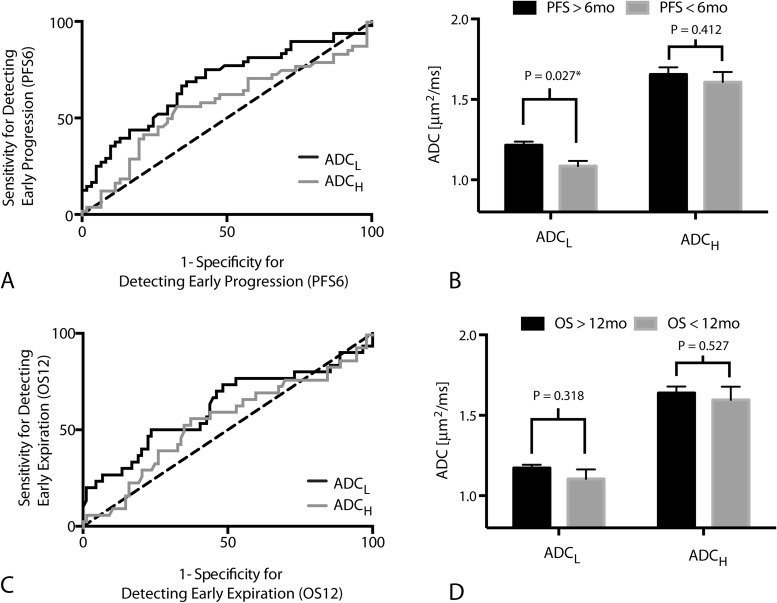
ROC curves. (A) ROC curve showing the sensitivity and specificity of ADC_L_ and ADC_H_ in detecting patients who progressed within 6 months of starting adjuvant TMZ (PFS6) (ADC_L_: ROC AUC = 0.6820 ± 0.05252 SEM, *P* = .0011; ADC_H_: ROC AUC = 0.5768 ± 0.057 SEM, *P* = .2187). (B) ADC_L_ and ADC_H_ measurements for individual tumors categorized based on progression before or after 6 months from the start of adjuvant TMZ (PFS6). Significant differences in ADC_L_ (*t* test, *P* = .027) but not ADC_H_ (*t* test, *P* = .412) were observed between patients progressing before and after 6 months from the start of adjuvant TMZ. (C) ROC curve showing the sensitivity and specificity of ADC_L_ and ADC_H_ in detecting patients who died within 12 months of starting adjuvant TMZ (OS12) (ADC_L_; ROC AUC = 0.6176 ± 0.06551 SEM, *P* = .0547; ADC_H_: ROC AUC = 0.55 ± 0.064 SEM, *P* = .4104). (D) ADC_L_ and ADC_H_ measurements for individual tumors categorized based on death before or after 12 months from the start of adjuvant TMZ (OS12). No significant differences were observed in measurements of ADC_L_ (*t* test, *P* = .318) or ADC_H_ (*t* test, *P* = .527) when patients were stratified by OS12.

Results suggest ADC_L_ is a significant predictor of patients that will progress within 6 months of starting adjuvant TMZ (Figure 3A; ROC AUC = 0.68 ± 0.053 SEM, *P* = .0011); however, ADC_H_ was not a significant predictor of progression by 6 months (Figure 2A; ROC AUC = 0.5768 ± 0.057 SEM, *P* = .2187). A threshold of ADC_L_ < 1.0 μm^2^/ms had a low sensitivity (34%) and high specificity (90%) for identifying patients that would progress within 6 months, meaning a high proportion of patients with low ADC_L_ after RT + TMZ will progress early after starting adjuvant TMZ (Figure 3B; *t* test, *P* = .027). (For reference, an ADC_L_ < 1.2 μm^2^/ms used in previous studies showed a sensitivity of 71% and specificity of 57% for PFS6.)

ADC_L_ also trended toward being a significant predictor of OS12 (Figure 3C; ROC AUC = 0.62 ± 0.066 SEM, *P* = .0547), whereas ADC_H_ did not predict OS12 (Figure 3C; ROC AUC = 0.55 ± 0.064 SEM, *P* = .4104). ADC_L_ showed a relatively low sensitivity (33%) but high specificity (82%) of predicting OS12 when using ADC_L_ < 1.0 μm^2^/ms for patient stratification. (For ADC_L_ < 1.2 μm^2^/ms, sensitivity/specificity = 73%/48%.)

### Progression-Free Survival

**Figure 4.**
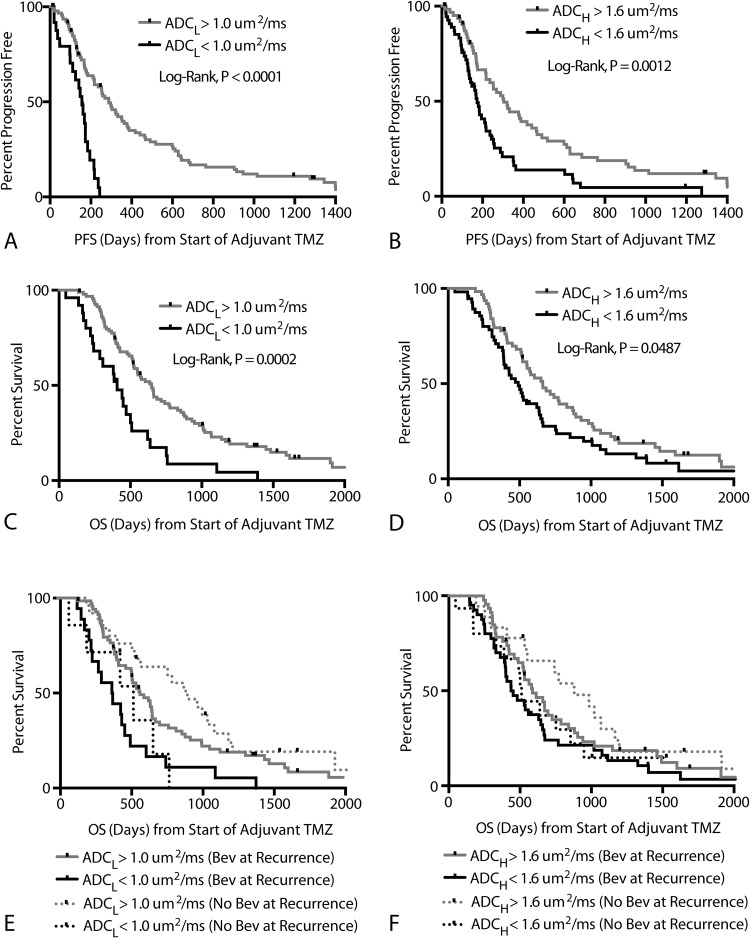
Progression-free and overall survival. (A) Kaplan-Meier curves showing significantly lower PFS in patients with ADC_L_ < 1.0 μm^2^/ms (log-rank, *P* < .0001; Cox multivariate, *P* = .0002). (B) Kaplan-Meier curves showing significantly lower PFS in patients with ADC_H_ < 1.6 μm^2^/ms in univariate analysis (log-rank, *P* = .0012); however, ADC_H_ was not significant in multivariate analysis (Cox multivariate, *P* = .9498). (C) Kaplan-Meier curves showing significantly lower OS in patients with ADC_L_ < 1.0 μm^2^/ms (log-rank, *P* = .0002; Cox multivariate, *P* = .0487). (D) Kaplan-Meier curves showing significantly lower OS in patients with ADC_H_ < 1.6 μm^2^/ms in univariate analysis (log-rank, *P* = .0487) but not when accounting for age and ADC_L_ (Cox multivariate, *P* = .5478). (E) Kaplan-Meier curves showing differences in OS based on ADC_L_ higher or lower than 1.0 μm^2^/ms for both bevacizumab-naïve (log-rank, *P* = .0130) and bevacizumab-treated (log-rank, *P* = .0029) patients at recurrence. No differences in OS were observed between patients treated with bevacizumab and those who were not within high ADC_L_ (log-rank, *P* = .1977) or low ADC_L_ (log-rank, *P* = .8959) groups. (F) Kaplan-Meier curves showing no differences in OS based on ADC_H_ higher or lower than 1.6 μm^2^/ms for both bevacizumab-naïve (log-rank, *P* = .1330) and bevacizumab-treated (log-rank, *P* = .1510) patients at recurrence.

Patients with an ADC_L_ < 1.0 μm^2^/ms had a significantly shorter PFS compared with the rest of the patients (Figure 4A; log-rank, *P* < .0001). Median PFS for patients exhibiting a low ADC_L_ (< 1.0 μm^2^/ms) was 156 days compared with a median PFS of 288 days for patients with a high ADC_L_ (> 1.0 μm^2^/ms). Similarly, patients with an ADC_H_ < 1.6 μm^2^/ms also demonstrated a significantly shorter PFS compared with the rest of the patients (Figure 4B; log-rank, *P* = .0012), with a median PFS of 173 days compared with 304 days for patients with an ADC_H_ > 1.6 μm^2^/ms. A Cox multivariate regression that examined the effects of age, ADC_L_, and ADC_H_ on PFS confirmed that ADC_L_ was a significant predictor of PFS (Cox: HR = 0.11 [95% CI: 0.03, 0.39], *P* = .0006) and age trended toward significance (Cox: HR = 1.02 [95% CI: 1.00, 1.04], *P* = .0521). No association between ADC_H_ and PFS was observed when accounting for age and ADC_L_ (Cox regression, *P* = .9498). No differences in ADC_L_ were observed between O6-methylguanine methyltransferase methylated and unmethylated tumors (*t* test, *P* = .38), but methylated tumors had significantly higher values of ADC_H_ (*t* test, *P* = .0443; mean ADC_H_ for methylated = 1.50 μm^2^/ms; mean ADC_H_ for unmethylated tumors = 1.72 μm^2^/ms).

### Overall Survival

Patients with an ADC_L_ < 1.0 μm^2^/ms had a significantly shorter OS compared with patients who had a higher ADC_L_ (Figure 4C; log-rank, *P* = .0002), with median OS for patients with a low ADC_L_ (< 1.0 μm^2^/ms) around 407 days compared with 648 days for patients with a high ADC_L_ (> 1.0 μm^2^/ms). Similarly, patients with an ADC_H_ < 1.6 μm^2^/ms also had a significantly shorter OS compared with patients who exhibited a higher ADC_H_ (Figure 4D; log-rank, *P* = .0487), with median OS for patients with a low ADC_H_ around 491 days compared with 662 days for patients with a high ADC_H_. Cox multivariate regression confirmed that both age (Cox: HR = 1.03 [95% CI: 1.01, 1.05], *P* = .001) and ADC_L_ (Cox: HR = 0.31 [95% CI: 0.09, 0.98], *P* = .047) were correlated with OS. ADC_H_ was not significantly associated with OS when accounting for age and ADC_L_ (Cox, *P* = .5478).

Significant OS differences were observed between high and low ADC_L_ in both patients who received bevacizumab at first recurrence (*n* = 87; log-rank, *P* = .003) and those who did not (*n* = 33; log-rank, *P* = .01) (Figure 4E). No differences were observed between high and low ADC_H_ in patients who received bevacizumab at first recurrence (log-rank, *P* = .1510) and those who did not (log-rank, *P* = .1330) (Figure 4F). No differences in OS were observed between patients treated with bevacizumab and those who were not within high ADC_L_ (log-rank, *P* = .20) or low ADC_L_ (log-rank, *P* = .90) groups.

## Discussion

Diffusion MRI measures of ADC have been shown to be correlated with both tumor cellularity (19, 23–25) and mitotic activity (26). Therefore, successful radiochemotherapy would be expected to result in a relatively higher amount of tumor cell destruction, leading to an increase in the diffusivity of water within the tumor as a result of the lack of restrictions to diffusion from structures such as cell membranes. Tumors with a low ADC following combined RT + TMZ, on the other hand, may consist of a more cellular, aggressive, possibly more treatment-resistant tumor phenotype. Results from this study support this hypothesis, suggesting patients with an ADC_L_ < 1.0 μm^2^/ms in contrast-enhancing tumors have a significantly shorter PFS and OS after starting adjuvant TMZ compared with other patients.

Previous studies using ADC histogram analysis have shown that tumor ADC_L_ values greater than 1.2 μm^2^/ms have a significantly longer PFS and OS in recurrent GBM treated with bevacizumab (13–15, 27). Using the threshold of 1.0 μm^2^/ms, we did not observe any difference in OS between patients treated with bevacizumab and those who were not. However, we did observe this trend at recurrence when using a threshold of 1.2 μm^2^/ms (data not shown), but this threshold was not significant when used for evaluating adjuvant TMZ and thus was not used in this study. Together, these results may suggest patients with a low ADC_L_ (< 1.0 μm^2^/ms) after RT + TMZ are likely to be nonresponsive to any subsequent therapies, including bevacizumab or additional chemotherapies; patients with a high ADC_L_ (> 1.2 μm^2^/ms) are likely to respond favorably to bevacizumab at first recurrence; and patients with an intermediate ADC_L_ (1.0 μm^2^/ms < ADC_L_ < 1.2 μm^2^/ms) may benefit from subsequent chemotherapy before treatment with bevacizumab.

There are a few limitations to this study that should be noted. It is important to point out that there was a potential selection bias because only patients who successfully completed surgical resection and RT + TMZ with a measurable contrast-enhancing tumor (>0.1 cc) were eligible for ADC histogram analysis. In addition, it is conceivable that some patients determined to have early progression after completing RT + TMZ actually had pseudoprogression, or treatment-related changes in vascular permeability that mimic radiographic changes similar to treatment failure or tumor growth. The addition of multimodal imaging techniques, including perfusion MRI (28), may have allowed for a more accurate delineation of pseudoprogression from true progression. Despite this potential confounding variable, we found significant differences in both PFS and OS in all patients based on diffusion characteristics as well as in patients with a PFS greater than 3 months from the end of RT + TMZ, where the incidence of pseudoprogression is likely to be highest. Moreover, this study involved acquiring diffusion MRIs using a variety of MRI systems and field strengths for the purpose of mimicking a clinical trial environment. Recent studies have shown that errors in ADC measurements vary nonlinearly from the scanner isocenter and that different MRI systems have different degrees of nonlinearity (29). Thus, this study would have benefited from the use of a temperature-controlled water phantom to account for system-specific errors in ADC measurements.

In summary, this study demonstrates that diffusion characteristics obtained using ADC histogram analysis can be used to predict PFS and OS after completing RT + TMZ and before adjuvant TMZ therapy. Results suggest that patients with an ADC_L_ < 1.0 μm^2^/ms are at increased risk for early progression and early death, indicating that ADC histogram analysis may be useful for patient-risk stratification after completing RT + TMZ. Future studies aimed at integrating ADC histogram analysis into clinical decision making as well as identifying biological correlates of diffusion characteristics are warranted.

**Conflicts of Interest:** Drs. Timothy F. Cloughesy, Albert Lai, Whitney B. Pope, and Benjamin M. Ellingson are paid consultants for Genentech, Inc., and Hoffman-La Roche, Ltd. Drs. Ellingson and Pope are also a paid consultant for MedQIA, LLC.
